# An Active Form of Sphingosine Kinase-1 Is Released in the Extracellular Medium as Component of Membrane Vesicles Shed by Two Human Tumor Cell Lines

**DOI:** 10.1155/2010/509329

**Published:** 2010-05-24

**Authors:** Salvatrice Rigogliuso, Chiara Donati, Donata Cassarà, Simona Taverna, Monica Salamone, Paola Bruni, Maria Letizia Vittorelli

**Affiliations:** ^1^Dipartimento di Biologia Cellulare e dello Sviluppo, Università di Palermo, Viale delle, Scienze ed. 16, 90128 Palermo, Italy; ^2^Dipartimento di Scienze Biochimiche, Università di Firenze, Viale G.B. Morgagni n. 50, 50134 Florence, Italy; ^3^Dipartimento Biopatologia e Metodologie Biomediche, Università di Palermo, Via Divisi 83, 90133 Palermo, Italy; ^4^IAMC-CNR, U.O. Capo Granitola, Mazara del Vallo, 91026 Trapani, Italy

## Abstract

Expression of sphingosine kinase-1 (SphK-1) correlates with a poor survival rate of tumor patients. This effect is probably due to the ability of SphK-1 to be released into the extracellular medium where it catalyzes the biosynthesis of sphingosine-1-phosphate (S1P), a signaling molecule endowed with profound proangiogenic effects. SphK-1 is a leaderless protein which is secreted by an unconventional mechanism. In this paper, we will show that in human hepatocarcinoma Sk-Hep1 cells, extracellular signaling is followed by targeting the enzyme to the cell surface and parallels targeting of FGF-2 to the budding vesicles. We will also show that SphK-1 is present in a catalitycally active form in vesicles shed by SK-Hep1 and human breast carcinoma 8701-BC cells. The enzyme substrate sphingosine is present in shed vesicles where it is produced by neutral ceramidase. Shed vesicles are therefore a site for S1P production in the extracellular medium and conceivably also within host cell following vesicle endocytosis.

## 1. Introduction

Malignant tumors have the remarkable ability to adapt their stromal environment to their benefit. They alter the surrounding extracellular matrix and modify normal cell behavior to facilitate tumor cell growth, invasion, immune evasion, and angiogenesis [1].

Most of these effects are mediated by the release of small vesicles from the tumor cells into the extracellular medium. Shed vesicles are known to facilitate tumor invasion [2–4], mainly by proteolytic enzymes associated with their membrane [5–9]. Indeed, the vesicle membranes are selectively enriched in some components including MMP-9 [7] and other proteolytic enzymes [4, 6], together with *β*1 Integrin and class I HLA molecules [7]. Enrichment of ganglioside G_D3_ and caveolin-1 has also been reported [10]. Moreover, vesicles use several mechanisms to contribute to tumor escape from immune reactions [11–16].

Notably, vesicles carry many proangiogenic growth factors, expressed differently depending on the vesicle origin, and that act on endothelial cells to promote angiogenesis. Indeed, FGF-2 was detected in vesicles shed by human hepatocarcinoma Sk-Hep1 cells [17, 18]; VEGF was found to be present in vesicles shed by human ovarian carcinoma cells [19] and in vesicles shed by neurons and astrocytes [20, 21]; angiogenin, IL-6, IL-8, VEGF, and TIMPs were found in vesicles shed by glioblastoma tumor cells [22]. Additionally, the sphingolipid fraction of vesicles shed by HT1080 fibrosarcoma and DU-145 human prostate carcinoma cells also showed proangiogenic activity [23]. Sphingomyelin is a normal component of plasma membranes where it is largely clustered in the outer membrane leaflet. It is subjected to intense metabolism which is responsible for the formation of a number of bioactive metabolites including ceramide, ceramide-1-phosphate, sphingosine, and sphingosine-1-phosphate (S1P) [24]. Ceramide, generated by sphingomyelinase (SMase) action on spingomyelin, appears to be a critical regulator of cell growth arrest, differentiation, and apoptosis [25, 26]. Sphingosine is formed by ceramide deacylation catalyzed by at least three different isoforms of ceramidase, which differ in optimal pH, primary structure, and cellular localization [27]. The enzyme sphingosine kinase (SphK) catalyzes the formation of S1P from sphingosine and ATP [28]. Two distinct SphK isoforms, SphK-1 and SphK-2, have been cloned [29, 30]. SphK-1, the more intensely researched isoform, is primarily localized in the cytosol, but, following ERK dependent phosphorylation elicited by various stimuli, it becomes translocated to the plasma membrane [31]. SphK-1 has been shown to regulate a wide variety of cellular processes, including the promotion of cell proliferation, survival, and motility [32] and, just as importantly, it possesses oncogenic potential [33]. Previous studies have established that SphK-1, like FGF-2 and several other proteins, can be released in the extracellular environment although it lacks a conventional secretory signal sequence.  The mechanism of SphK-1 secretion is unconventional and likely involves a nonstandard pathway independent of the endoplasmic reticulum/Golgi system; the SphK-1 secretion mechanism is only known to require functional actin dynamics [34]. Notably, the SphK product S1P, among multiple biological activities, exerts a strong proangiogenic effect which is known to act synergistically with growth factors such as FGF-2 [35, 36] and VEGF [35]. 

In this study we investigated whether vesicles shed by hepatocarcinoma and carcinoma cultured cells contain S1P-generating enzymes. The data from this research demonstrates that neutral ceramidase (nCDase) and SphK-1 are localized in vesicles, supporting the view that S1P participates in the proangiogenic activity exerted by these particles.

## 2. Materials and Methods

### 2.1. Cells and Culture Media

Human SK-Hep1 hepatocarcinoma cells were grown in Dulbecco's modified Eagle's medium supplemented with 10% fetal calf serum (FCS; Euroclone, Celbio). Human breast carcinoma 8701-BC cells, kindly provided by Profcessor Minafra [37], were grown in RPMI 1640 supplemented with 10% fetal calf serum (FCS; Euroclone, Celbio). Bovine GM7373 fetal aortic endothelial cells were grown in Eagle's minimal essential medium (Euroclone, Celbio) supplemented with 10% FCS, vitamins, and essential and nonessential amino acids.

### 2.2. Cell Extraction

Cells were removed from plate by a scraper and centrifuged at 2000 g for 5 minutes; pelleted cells were then resuspended in 300 *μ*L of Triton X100 (1%) on phosphate buffer saline (PBS). After 10- minute incubation at room temperature, the cell extract was centrifuged at 800 g for 10 minutes The amount of protein extracted from cells was determined using the Bradford microassay method (Bio-Rad, Segrate, Milan, Italy) employing bovine serum albumin (Sigma-Aldrich) as a standard.

### 2.3. Vesicle Purification from Conditioned Medium

Vesicles were purified from the conditioned medium as described above [38]. Briefly, the medium was conditioned by culturing subconfluent healthy cells for 3 or 24 hours and were centrifuged at 2000 g for 10 minutes and at 4000 g for 15 minutes The supernatant was ultracentrifuged at 105,000 g in a Ti-60 Rotor (Backman) for 90 minutes Pelleted vesicles were resuspended in PBS. The amount of isolated vesicles was determined by measuring the protein concentration using the Bradford microassay method (Bio-Rad, Segrate, Milan, Italy) using bovine serum albumin (Sigma-Aldrich) as a standard.

### 2.4. Western Blotting

After SDS-PAGE electrophoresis were cast in 7.5% gels, proteins was blotted onto a nitrocellulose membrane (Hybond; Amersham Biosciences) that was saturated with 3% nonfat dry milk in Tris Buffer Saline 50 mM pH  7.9/Tween 0.05% (TBS-T). After 5 washes in TBS-T for 5 minutes each, the nitrocellulose membranes were incubated overnight at 4°C, with mouse monoclonal anti-nCDase antibody 1 : 200 (kindly donated by Professor Ito, Fukuoka, Japan) [39]. The primary antibody was followed by peroxidase-conjugated anti-mouse antibodies (1 : 10000) (Amersham Biosciences) for 1 hour at room temperature. Immunocomplexes were visualized with the ECL Western blotting kit (Amersham Biosciences) using Hyperfilm.

### 2.5. Confocal Immunofluorescence

Cells, seeded at low density (2.000 cells/well) onto microscope cover slips in 12-well culture plates (Nunc), were grown overnight in the complete medium and, when needed, for 3 more days in a serum-free medium with three medium changes. SphK-1 and SphK-2 were detected by using as primary antibodies, rabbit polyclonal anti-SphK-1 antibody (kindly donated by Prof. Obeid, Charleston, SC, USA) [40] 1 : 100, and rabbit polyclonal anti-SphK-2 antibodies 1 : 100 (kindly provided by Dr. Nakamura, Kobe, Japan), respectively, [41]. Secondary antibodies used were antirabbit TRITC-conjugated antibodies (1 : 200 Sigma); *β*_1_ Integrin was detected using C27 anti-*β*_1_ Integrin rat primary monoclonal antibody 1 : 150 [42] and antirat TRITC conjugated secondary antibody (1 : 320, Sigma). FGF-2 was detected using mouse monoclonal anti-FGF-2 antibody (0.5 mg/mL 1 : 200, Upstate Biotechnology type II) and Texas Redconjugated antimouse antibody (1 : 200, Amersham Biosciences).

In order to stain nuclei, cells were fixed in 3.7% formaldehyde and then stained for 10 minutes with propidium iodide (Sigma).

Immunostained cells were analyzed by confocal microscopy (Olympus 1X70 with Melles Griot laser system).

### 2.6. Staining of Vesicle Lipids

Vesicle lipids were stained with the lipophilic styryl compound FM4-64 (Molecular Probes). Purified vesicles (180 *μ*g) were resuspended in 1 ml PBS and stained with FM4-64 dissolved in PBS without calcium and magnesium. FM4-64 was added at a final concentration of 5 *μ*g/ml; samples were incubated at room temperature for 15 minutes. Stained vesicles were collected by centrifugation at 50,000 g for 1 hour, resuspended in 50 *μ*l PBS and added to GM7373 cells to monitor vesicle targeting.

### 2.7. Transient Cell Transfection

SK-Hep1 cells were plated in six-well culture plates at 3 × 10^5^ cells/well and maintained overnight in high-glucose DMEM containing 10% fetal calf serum, 2 mM L-glutamine, 100 IU/ml penicillin, and 100 *μ*g/ml streptomycin. The next day, cells were transfected with SKpeGFP plasmid encoding for SphK-GFP chimera (kindly donated by Professor Spiegel, Richmond, VA, USA) [43]. Transfection was carried out using Lipofectamine Reagent (GIBCO Life Technologies), according to the manufacturer's instructions. 

### 2.8. Sphingosine Kinase Assay

SphK activity was assayed in isolated vesicles or serum-free conditioned medium as described by Olivera et al. [28]. Briefly, 50 *μ*g vesicle proteins were resuspended in 100 *μ*l of the reaction mixture which contained 20 mM Tris-HCl, pH  7.4, 20% (v/v) glycerol, 1 mM *β*-mercaptoethanol, 1 mM EDTA, 1 mM sodium orthovanadate, 15 mM sodium fluoride, protease inhibitors (10 mg/ml leupeptin,10 mg/ml aprotinin, and 1 mM PMSF), and 0.5 mM 4-deoxypyridoxine.

The serum-free conditioned medium was concentrated approximately 40-fold before being employed for enzymatic activity measurement.

The enzymatic reaction was initiated by adding 50 *μ*M sphingosine and 1 mM [*γ* ^32^P] ATP. In some cases, assays were performed which omitted sphingosine to evaluate the availability of endogenous sphingosine. After 30- minutes incubation at 37°C, the reaction was terminated by adding 20 *μ*l 1 N HCl and 900 *μ*l of chloroform/methanol/HCl (100 : 200 : 1, v/v). Lipids were then extracted, separated by TLC, labeled SIP and quantified by liquid scintillation essentially as previously described [44]. Specific activity of SphK was expressed as pmol of S1P produced/min/mg of protein.

### 2.9. Neutral Ceramidase Activity Assay

NCDase activity was determined using C12-NBD-ceramide as a substrate as previously described [45]. Briefly, 100 pmol of C12-NBD-ceramide (NBD-C12:0, d18:1) was incubated for 2 h at 37°C with an appropriate amount of proteins in 20 *μ*l of 25 mM Tris-HCl buffer  pH  7.5 and 0.25% (w/v) Triton X-100. Samples were then applied to a TLC plate, which was developed with chloroform, methanol, and 25% ammonia (90 : 20: 0.5, v/v). Spots corresponding to NBD-dodecanoic acid and C12-NBD-ceramide were scraped, incubated with methanol at 37°C to extract the compounds from the silica, and their fluorescence at 470/525 nm excitation/emission wavelengths was measured using a Shimadzu 9000 spectrophotofluorimeter. The compounds were quantified using a standard curve of known amounts of C12-NBD-ceramide and NBD-dodecanoic acid. 

## 3. Results

### 3.1. Immunolocalization of SphK-1 and Sphk-2 in 8701-BC and Sk-Hep-1 Cells

**Figure 1:**
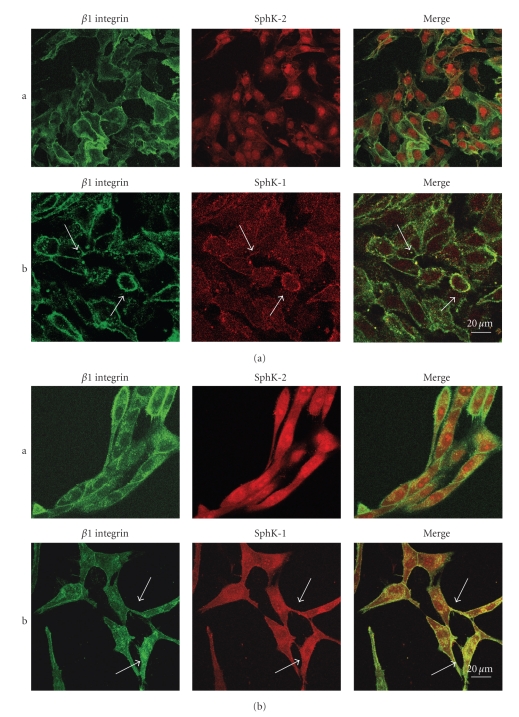
Comparative analysis of   *β*1 Integrin, SphK-2, and SphK-1 immunolocalization. (a) Localization in 8701-BC cells. (b) Localization in Sk-Hep1 cells. Line a: Immunolocalization of *β*1 Integrin and SphK-2 showing a different distribution of the two molecules. Line b: Immunolocalization of *β*1 Integrin and SphK-1. Arrows indicate colocalization areas.   *β*1 Integrin was detected using FITC-conjugated secondary antibodies and SphK-2 and SphK-1 using Texas red-conjugated secondary antibodies. Arrows indicate colocalization areas.

In a first group of experiments, expression and localization of SphK-1 and SphK-2 were analyzed by immunofluorescence in 8701 BC carcinoma cells and in Sk-Hep1 hepatocarcinoma cells (Figure 1).

Moreover, since it had been previously demonstrated that *β*_1_ integrin is clustered in shed vesicles [7, 17], distribution of the two proteins was compared with distribution of *β*_1_ integrin. 

**Figure 2:**
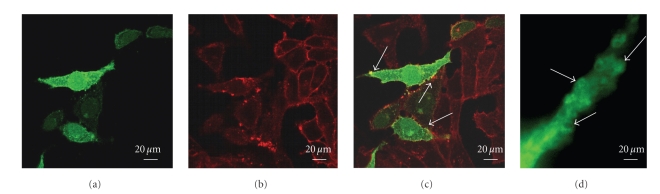
Comparative analysis of SphK-1 and of *β*1 Integrin localization in transfected Sk-Hep1 cells. (a) GFP-bound SphK-1 localization in transfected cells. The protein is localized in cell membranes where it shows uneven clustering in small spots. (b) Immunolocalization of  *β*1 Integrin detected using Texas red-conjugated secondary antibodies. *β*1 Integrin is seen in cell membranes of both transfected and non-transfected cells. Like SphK-1, *β*1 Integrin shows uneven clustering in small spots. (c) Double staining shows colocalization of the two proteins in some areas of the plasma membrane (indicated by arrows). (d) Enlargement of a cell protrusion showing budding areas (indicated by arrows) in which SphK-1 appears to have a preferential localization.

In both cell lines, the distribution of SphK-2 was quite different from the distribution of *β*_1_ integrin (Figures 1(a) and 1(b) line a). SphK-2 was indeed clustered in the cell nucleus. However, it was absent in the cell membrane where instead *β*_1_ integrin was located. In contrast, SphK-1 and *β*_1_ integrin appear to colocalize at the plasma membrane (Figures 1(a) and 1(b) line b). Moreover, as can more clearly be seen in cells transiently transfected with GFP-SphK-1 (Figure 2), both SphK-1 and *β*_1_ integrin seem to be more dense in specific areas of the plasma membrane, and clustering appears to occur in areas of the cell membrane from which vesicles are released (Figure 2(d)).

### 3.2. Effects of Serum Addition on SphK-1 Trafficking toward the Cell Periphery

In a previous study, we observed that vesicles shed by Sk-Hep1 cells mediate FGF-2 release and that vesicle shedding and release of FGF-2 were simultaneously induced by the addition of serum to starved cells [17]. By monitoring intracellular movements of the growth factor subsequent to serum addition, we showed that within one hour FGF-2 was targeted to the cell periphery and to the cell nucleus and nucleolus. FGF-2 movements toward the cell periphery required actin filament integrity [18]. 

Similarly to FGF-2, SphK-1 is a leaderless protein secreted by unconventional mechanisms [29, 34] whose movement toward the cell periphery is mediated by actin filaments [34]. We therefore theorized that the two proteins could share a similar export mechanism and we analyzed whether intracellular movements of SphK-1 were influenced by serum addition and whether the enzyme colocalized with FGF-2.

**Figure 3:**
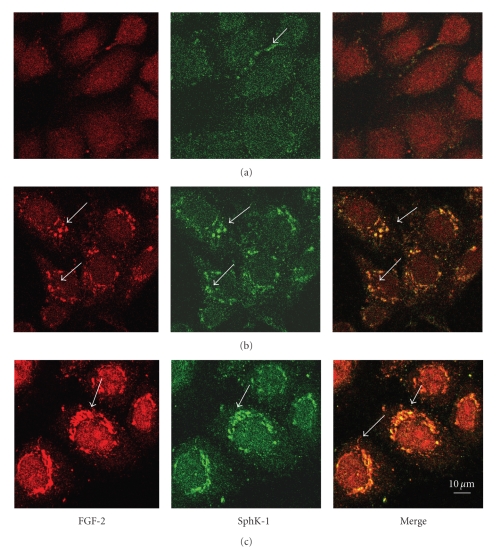
Course of endogenous SphK-1 and endogenous FGF-2 targeting to the cell periphery over time, observed by immunolocalization experiments. FGF-2 and SphK-1 immunolocalization at 0, 30, and 60 minutes after serum addition (lines (a), (b), (c), resp.) Sections 3 *μ*m from surface. FGF-2 was detected using Texas red-conjugated secondary antibodies; SphK-1 was detected using FITC-conjugated antibodies. Arrows indicate granules of protein localization.

Intracellular distribution of SphK-1 was therefore analyzed by immunolocalization in starved cells as well as at time intervals after serum addition and compared with FGF-2 distribution. As shown in Figure 3, in starved cells the two proteins did not colocalize. In SphK-1 they were partially localized in small granules and in FGF-2 they were totally dispersed.

Thirty minutes after the serum was added both proteins were clustered in granules and showed a clear colocalization. One hour after the serum was added large granules containing both proteins were present near the cell membrane. In contrast, cell nuclei were exclusively stained by anti-FGF-2 antibodies. 

These results suggest that FGF-2 and SphK-1 share a similar transport mechanism toward the cell periphery and that the two proteins are both likely to be targeted to the budding vesicles.

### 3.3. Detection of an Active Form of SphK-1 in Shed Vesicles

In order to establish if an active form of SphK-1 is shed as a component of membrane vesicles, we ascertained SphK activity in vesicles or cell-conditioned media in some experiments. Results reported in Table 1 show that SphK-1 was clearly detectable in vesicles shed by Sk-Hep1 and 8701-BC cells, although the activity was found to be greater in vesicles shed by Sk-Hep1 cells. 

SphK-1 activity was also tested in vesicle-deprived conditioned media. No enzymatic activity could be detected in serum-containing media, even when it was concentrated. Instead, it was possible to detect a low SphK activity in 40-fold-concentrated serum-free medium that had been conditioned by maintaining cells in culture for 24 h. As shown in Table 1, when vesicles were collected from a medium to which 2M NaCl had been added in order to solubilize proteoglycans and other molecules unspecifically bound to the vesicle membrane, the enzymatic activity was not affected. 

The addition of sphingosine increased labeled S1P production; however, the catalytic activity of the enzyme was also observed when this substrate was not added. This result indicates that sphingosine is already present in shed components of membrane vesicles.

**Figure 4:**
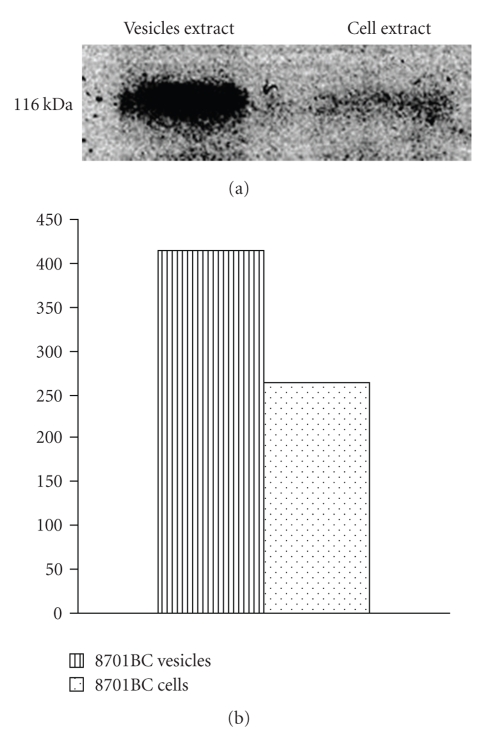
Western Blot analysis for nCDase (a) and its densitometric analisys (b) on 8701BC vesicles and cell extracts.

Sphingosine is likely produced by nCDase, which is known to be localized in plasma membranes and also found in the extracellular medium [46, 47]. An enzymatically active form of nCDase was actually found to be present in vesicles shed by 8701 BC (Figure 4 and Table 2). Moreover, in Sk-Hep1 cells, we observed colocalization of nCDase and *β*_1_ integrin (data not shown), indicating that it is also likely that nCDase is present in vesicles shed by this cell line.

### 3.4. Fate of Shed Vesicles

Since S1P can be produced in the membrane of shed vesicles, the molecule could remain, at least in part, within the vesicle. Vesicles could adhere to the plasma membrane of cells surrounding the tumor and S1P could exert its effects by interacting with receptors localized at the cell surface. On the other hand, vesicles could be internalized by the host cell and consequently release S1P inside cells where it could act as intracellular messenger. In order to verify these hypotheses, we analyzed the targets of shed vesicles after adding them to GM7373 cells, an immortalized line of embryonic bovine aortic endothelial cells.

**Figure 5:**
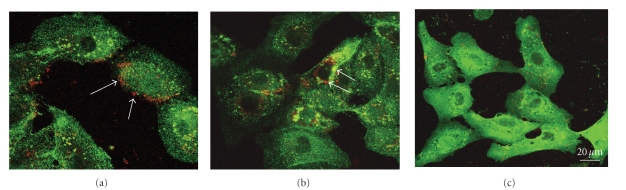
Interactions of shed vesicles with endothelial cells. Vesicles shed by SK-Hep1 cells, labelled with lipid styryl dye FM4-64 (red fluorescence), were added to GM7373 endothelial cells in which *β*1 Integrin was stained using FITC-conjugated secondary antibodies (green fluorescence). Cells were incubated with vesicles, respectively, for (a) 10 minutes, (b) 20 minutes, and (c) 30 minutes. The arrows indicate vesicle localization.

 For this purpose, vesicles released by Sk-Hep1 cells were labeled for 15 minutes with the lipid marker FM4-64. Labeled vesicles were then added to in vitro cultured GM7373 cells which in turn had been labeled with antibodies against *β*_1_ integrin. As shown in Figure 5, at 10 minutes after incubation vesicles were observed to be bound to the cell membrane, while after 20-minutes incubation most vesicles were internalized and visible in the cytoplasm. At 30 minutes, the signal borne by lipid marker FM4-64 was no longer visible, indicating that the lipids of the vesicle membranes had degraded. In principle therefore, vesicle-associated S1P could act on both membrane receptors and intracellular targets.

## 4. Conclusions

Membrane vesicles shed by tumor cells appear to exert a variety of effects on the surrounding cells. Vesicles are rich in enzymatic activities able to modify extracellular medium composition, thus facilitating tumor cell migration and angiogenesis. Depending on their origin, they also convey different signaling molecules which exert their effects on lymphocytes, mesenchymal cells, and endothelial cells. Shed vesicles have been shown to induce angiogenesis using a variety of mechanisms including the action of proteins such as FGF-2, VEGF, angiogenin, IL-6, IL-8, and TIMPs, and lipid molecules such as sphingomyelin. 

Based on the present results, nCDase and SphK-1 can now be included among the signaling molecules transferred by shed membrane vesicles, suggesting that S1P formed at level of the vesicle membranes plays a role in the biological processes regulated by these particles.

Interestingly, nCDase, here identified as a component of shed vesicles, was previously identified in various subcellular compartments such as endosomes, mitochondria, and microdomains of the plasma membrane [39, 48] but was also found to be involved in extracellular sphingolipid metabolism [47]. In this regard it was demonstrated that although nCDase is localized at the plasma membrane as a type II integral membrane protein, the enzyme is released in the extracellular medium after the proteolytic action of secretases [49, 50]. Moreover, in agreement with the present results, nCDase, together with acid SMase, was identified as a component of a complex in the cell membrane domain which is subjected to budding as well as in conditioned medium associated with caveolin-1, a key structural protein of caveole [51] which was also detected in shed vesicles [52]. SphK-1 is a secreted leaderless protein, and the shedding of membrane vesicles appears to represent a mechanism which accounts for its secretion. The presence of the enzymatic protein in shed vesicles does not per se exclude the face that other mechanisms may also participate in the release of SphK-1. Indeed it was reported that, in FGF-1 overexpressing NIH 3T3 cells, SphK-1 is secreted together with FGF-1 as a component of a high molecular weight complex [53, 54]. However, SphK-1 is also secreted by cells which do not express FGF-1, and in the absence of stress signaling which induces FGF-1 secretion. Here we have demonstrated that at least in some instances SphK-1 is secreted as a component of shed vesicles. Since shed vesicles also contain nCDase, which provides the rate-limiting substrate for S1P production by catalyzing sphingosine generation, it is likely that these particles cause sustained S1P production.

SphK-1 and S1P produced by its enzymatic activity are able to mediate a network of paracrine signaling. It is well known that acting on the two membrane receptors SIP_1_ and SIP_3_, S1P induces morphogenesis in HUVEC cells [35]. Moreover, since vesicles carry several other molecules able to affect angiogenesis, the overall effects of vesicles on surrounding endothelial cells will be amplified and differently modulated depending on the specific composition of the vesicles.

The exact mechanism by which the S1P message borne by shed vesicles is delivered to the host cell remains to be explored. Indeed, an attractive hypothesis is that after interacting with the recipient cell plasma membrane, vesicles are internalized via endocytosis. SphK-1 and SIP would therefore be delivered into the cytoplasm of the receiving cell, where, as already known, SIP could exert its intracellular effects, regulating various processes among which cell survival is prominent [55, 56]. Indeed vesicles were shown to convey molecules, such as mRNA and iRNA, to the cytoplasm of surrounding cells, and it was recently reported that mRNA included in shed vesicles can be translated in recipient cells following endocytosis [22].

Alternatively, because SphK-1 association with cell membranes is strengthened by interaction with phosphatidylserine following its phosphorylation [57] and since exposure of phosphatidylserine on the outer leaflet is a hallmark of shed membrane vesicles [58, 59], it can also be speculated that SphK-1, localized in these particles, can generate S1P in the extracellular environment. If this is the case, the bioactive lipid generated outside the endothelial cells could determine key effects on development and proliferation of endothelial cells, acting as a ligand of S1P_1_ and/or S1P_3_ receptors [60, 61]. Independent of the action mechanism, the ability of shed vesicles to carry on key enzymes for S1P production which this study brought to light illuminates a novel aspect of their biochemical properties which is relevant to a complete understanding of their proangiogenic activity.

## Figures and Tables

**Table 1:** Enzimatic assay of SphK-1 activity.

Sample	Incubation mixture with exogenous sphingosine	Incubation mixture without exogenous sphingosine
SK-Hep1 vesicles	43.93 pmol/min/mg of protein	26.70 pmol/min/mg of protein
SK-Hep1 vesicles*	43.59 pmol/min/mg of protein	
SK-Hep1 C.M.	2.23 pmol/min/mg of protein	
8701 BC vesicles	14.44 pmol/min/mg of protein	12.61 pmol/min/mg of protein
8701 BC vesicles*	16.70 pmol/min/mg of protein	
8701 BC CM	2.16 pmol/min/mg of protein	

*Vesicles recovered from medium in which 2 M NaCl had been added.

**Table 2:** 

Sample	Enzimatic activity of nCDase
8701 BC cells	2,08 pmol/min/mg of protein
8701 BC vesicles	5,25 pmol/min/mg of protein
